# Assessing Digital Risk in Psychiatric Patients: Mixed Methods Study of Psychiatry Trainees’ Experiences, Views, and Understanding

**DOI:** 10.2196/19008

**Published:** 2020-07-29

**Authors:** Golnar Aref-Adib, Gabriella Landy, Michelle Eskinazi, Andrew Sommerlad, Nicola Morant, Sonia Johnson, Richard Graham, David Osborn, Alexandra Pitman

**Affiliations:** 1 Division of Psychiatry University College London London United Kingdom; 2 Camden and Islington NHS Foundation Trust London United Kingdom; 3 The Tavistock and Portman NHS Foundation Trust London United Kingdom; 4 Good Thinking: the London Digital Mental Well-being Service Mental Health Programme London United Kingdom

**Keywords:** risk assessment, internet, suicide, self-injurious behavior, mental health, psychiatrists, mixed methods, mobile phone

## Abstract

**Background:**

The use of digital technology can help people access information and provide support for their mental health problems, but it can also expose them to risk, such as bullying or prosuicide websites. It may be important to consider internet-related risk behavior (digital risk) within a generic psychiatric risk assessment, but no studies have explored the practice or acceptability of this among psychiatrists.

**Objective:**

This study aimed to explore psychiatry trainees’ experiences, views, and understanding of digital risk in psychiatry. We predicted that clinician awareness would be highest among trainees who work in child and adolescent mental health services.

**Methods:**

We conducted a cross-sectional survey of psychiatry trainees attending a UK regional trainees’ conference to investigate how they routinely assess patients’ internet use and related risk of harm and their experience and confidence in assessing these risks. We conducted focus groups to further explore trainees’ understandings and experiences of digital risk assessment. Descriptive statistics and chi-squared tests were used to present the quantitative data. A thematic analysis was used to identify the key themes in the qualitative data set.

**Results:**

The cross-sectional survey was completed by 113 out of 312 psychiatry trainees (response rate 36.2%), from a range of subspecialties and experience levels. Half of the trainees (57/113, 50.4%) reported treating patients exposed to digital risk, particularly trainees subspecializing in child and adolescent psychiatry (17/22, 77% vs 40/91, 44%;*P*=.02). However, 67.3% (76/113) reported not feeling competent to assess digital risk. Child and adolescent psychiatrists were more likely than others to ask patients routinely about specific digital risk domains, including reckless web-based behavior (18/20, 90% vs 54/82, 66%; *P*=.03), prosuicide websites (20/21, 95% vs 57/81, 70%; *P*=.01), and online sexual behavior (17/21, 81% vs 44/81, 54%; *P*=.02). Although 84.1% (95/113) of the participants reported using a proforma to record general risk assessment, only 5% (5/95) of these participants prompted an assessment of internet use. Only 9.7% (11/113) of the trainees had received digital risk training, and 73.5% (83/113) reported that they would value this. Our thematic analysis of transcripts from 3 focus groups (comprising 11 trainees) identified 2 main themes: barriers to assessment and management of digital risk, and the double-edged sword of web use. Barriers reported included the novelty and complexity of the internet, a lack of confidence and guidance in addressing internet use directly, and ongoing tension between assessment and privacy.

**Conclusions:**

Although it is common for psychiatrists to encounter patients subject to digital risk, trainee psychiatrists lack competence and confidence in their assessment. Training in digital risk and the inclusion of prompts in standardized risk proformas would promote good clinical practice and prevent a potential blind spot in general risk assessment.

## Introduction

### Background

The use of digital technology continues to rise, with an estimated 4.13 billion internet users worldwide [1]. The United Kingdom has the second highest number of internet users in Europe [2], and most people in the United Kingdom own a smartphone, with over 95% of 16- to 24-year-olds owning one [3]. The use of web-based social networks is also increasing rapidly; Facebook had 1.62 billion daily active users by the end of 2019 [4,5]. The rising use of digital technology poses new risks for patients and thus is a complex challenge for psychiatrists. Although it has benefits in terms of social communication, peer support, self-management, and dissemination of knowledge, it can also expose people to adverse novel activities, which are harmful to themselves and others.

Harms related to internet-related behaviors (which we refer to as *digital risk*) have not yet been quantified in the general population or for those with mental health problems. A classification system has been proposed for digital risk in children and families and includes commercial, aggressive, and sexual risks and corruption of values. These are further subclassified by 3 modes of web-based communication: person as recipient, person as participant, and person as initiator [6]. Although this classification system was developed for children and families, it is also relevant to both young people and adults who can be vulnerable to risk in similar domains. The classification of digital risk (adapted from the European research on children’s internet use [6]) has been described in Table 1.

**Table 1:** Classification of digital risk.

Risks	Content (person as recipient)	Contact (person as participant)	Conduct (person as initiator)
Commercial	Advertising, spam	Tracking/harvesting personal information	Gambling, illegal downloads, hacking
Aggressive	Violent/gruesome/hateful content	Being bullied, harassed, or stalked	Bullying or harassing another
Sexual	Pornographic/harmful sexual content	Meeting strangers, being groomed	Creating/uploading pornographic material
Values	Racist, biased information/advice (eg, drugs)	Self-harm, unwelcome persuasion/coercion	Providing advice (eg, suicide/proanorexia)

Increasing internet use has been accompanied by a rise in discriminatory or criminal activity on social media platforms, such as grooming, cyberbullying, and harassment, with over 32,000 Facebook crimes reported to the police in the United Kingdom in 2019 [7]. Approximately one-fifth of US adolescents report experiences of cyberbullying (bullying or harassment using electronic forms of contact) [8], most frequently via a social media site [9]. The frequency of cyberbullying is associated with self-harm, depression, anxiety, suicidality, and drug and alcohol problems [8,10-12]. A multicenter interview study of 11- to 16-year-olds across 25 European countries used weighted estimates to suggest that 17% of children have been exposed to sexual images on the web (ie, images or video of someone naked, of their genitals, of someone having sex, and showing sex in a violent way and other sexual images), 13% to proanorexia websites, and 20% to websites that publish hate messages [13].

Other internet-related risks include prosuicide websites containing advice and a discussion of lethal methods. A UK cross-sectional study of young adults found that suicide-/self-harm–related internet use was reported by 22.5% of participants, with both prosuicide and suicide prevention sites being accessed [14]. Another study in both children and adults found that self-harm related to internet use was associated with high suicidal intent [15]. The widespread availability of prescriptions and illicit medication on the web is also concerning. There are reports of web-based pharmacies that employ no restrictions on the age of consumers, where products can be bought in large quantities without a prescription and some preparations could contain hidden toxic compounds [16]. With increasing numbers of people obtaining medication and other products on the web, the risk of intentional or unintentional toxicity is potentially increased [17].

People with mental illness have been shown to have higher levels of internet addiction, and there are positive and significant associations with attention deficit hyperactivity disorder, depression, and anxiety [14]. They may also be more vulnerable to prosuicide and other potentially harmful websites [18].

These risks also apply to the elderly, who are relatively technologically naive, and to children, who as *digital natives* [19] *have been brought up with much greater exposure to the internet via computers and mobile devices during a vulnerable stage in their development*. Both are vulnerable to exploitation; the former because they may not be aware of scammers’ techniques, and the latter because they are less likely to have emotional resources or experience to counter exploitation or other risks. Of particular concern are those in each age group who have mental health problems, who are already vulnerable to exploitation offline and for whom this risk is amplified in their online life. Young people who are vulnerable offline in relation to social disadvantage are more likely to report high-risk web experiences relating to inappropriate content, conduct, contacts, and cyber scams [20]. Young people are also more susceptible than adults to the effects of social modeling of suicidal behavior, including in relation to the media [21,22].

Health care professionals working in children’s services are likely to encounter patients with exposure to such risks and may therefore have more awareness and expertise in assessment and management.

At present, there is no accepted protocol for assessing digital risk and no existing validated questionnaire for assessing digital risk. To our knowledge, there has been no research internationally exploring the practice of digital risk assessment among mental health professionals. Consequently, this study was designed by psychiatry trainees to investigate the practice of assessment and management of online risk.

### Objectives

This study aimed to explore experiences, views, and understanding of patients’ digital risk among trainee psychiatrists (doctors specializing in psychiatry training with up to 6 years of experience in working in psychiatric services) in the United Kingdom. We predicted that clinician awareness would be low but highest among trainees who work in child and adolescent mental health services (CAMHS), as these psychiatrists care for patients with more active digital engagement. We also aimed to examine experiences of training in digital risk and existing risk assessment practices.

## Methods

We conducted a sequential mixed methods study of psychiatry trainees’ views and experiences, using a cross-sectional survey followed by a series of focus groups to generate in-depth qualitative understanding.

### Cross-Sectional Survey

#### Survey Instrument

We developed a survey tool for this study in 3 stages. First, we reviewed the literature on digital risk, including the *EU Kids* online report [23] and media reports of digital risk, to generate a theoretical framework for digital risk. We then drafted a questionnaire with input from clinicians and specialists from the UK National Health Service (NHS) mental health service and the Tavistock and Portman NHS Trust. We presented our draft survey to the London Digital Safety Network meeting, a forum of mental health professionals who are leading experts in digital risk in psychiatry, to assess face validity and revise it in response to their feedback. Finally, the questionnaire was piloted in 2 stages, initially as a paper survey with a group of 20 trainee psychiatrists and then, following revisions, a larger pilot of 120 mental health professionals (on paper or via a website address). We again amended the questionnaire based on their feedback.

The final draft of the questionnaire included specific questions about trainees’ clinical experience of, and training in, digital risk (Multimedia Appendix 1). It obtained demographic information and occupational history, including training grade (core trainee [up to 3 years of psychiatry experience rotating across a broad range of specialties within psychiatry], higher trainee [more than 3 years of psychiatry experience with specialism in their patient group, eg, general adults or children and adolescents], or others [other training grades]); duration of experience in psychiatry; psychiatry specialty (children and adolescents [aged 18 years and below]; working age adults [aged 18 to 64 years], older adults [aged 65 years and above], forensic patients, or others [eg, intellectual disability psychiatry or specialist psychotherapy services]); and the respondent’s own frequency of internet use and ownership of specific devices and relevant technology.

#### Data Collection

Using this questionnaire, we conducted a cross-sectional survey of trainee psychiatrists attending a conference in London, United Kingdom, in November 2013. All London-based psychiatry trainees were expected to attend. We distributed paper copies of the survey, which could be completed anonymously, and provided access to a web-based version to every attendee. There were no financial incentives for completing the survey. We provided written information at the start of the survey including the length of the survey, and completion of the survey indicated informed consent. We sent reminders after 2 and 4 weeks to all of the attendees at the conference to prompt those who had not yet completed the survey.

Ethical approval was not required by the psychiatry training committee, as the survey was regarded as a service improvement project.

#### Statistical Analysis

We used descriptive statistics to describe demographic data, occupational history, digital technology use and nature, extent of digital risk assessment experience, and reported confidence and competence in digital risk assessment. We compared trainees specializing in child and adolescent psychiatry with those from other subspecialties (general adult, forensic, learning disability, older adults, substance misuse, psychotherapy, academic, and liaison) using chi-square tests and Mantel-Haenszel tests. A *P* value of .05 was used as the threshold for statistical significance. We conducted all analyses using SPSS for Windows, version 22.

### Focus Groups

#### Setting and Participants

We conducted focus groups with London-based trainee psychiatrists from January 2015 to June 2016. The participants were purposively sampled from 2 training programs within the London area. This was not a nested sample from those who had taken part in the earlier regional conference, but participation in both was not precluded.

We sent emails to all potential participants via the training program’s administrators inviting them to contact us if interested in taking part. We included participants who are training in psychiatry within North and Central London training schemes. We used purposive sampling based on criteria of stages of training (core vs higher training; years of experience) and subspecialties to ensure broad representation of trainees and gather a wide range of opinions, experiences, and perspectives.

Ethical permission for focus group data collection and analysis was obtained from the University College London Research Ethics Committee (Project ID: 6589/001).

#### Data Collection

We anticipated reaching data saturation once 10 to 20 trainee psychiatrists had participated in focus groups and aimed to conduct 3 focus groups with 3 to 6 participants per group. To encourage disclosure among less experienced trainees, we grouped participants by training, so that 1 focus group comprised core trainees (1-3 years of psychiatry experience), 1 comprised higher trainees in general adult psychiatry (more than 4 years of psychiatry experience specializing in working age patients), and 1 comprised higher trainees in CAMHS (4-7 years of psychiatry experience with specialization in children aged under 18 years).

Focus groups were conducted at times adjacent to regular teaching commitments, or during lunch breaks, for participants’ convenience. Each focus group lasted approximately 50 min and was facilitated by 2 researchers, who were themselves psychiatric trainees (1 in general adult psychiatry [GA] and 1 in older adult psychiatry [AS]). The focus groups were audio recorded and transcribed verbatim by the first author. All participants provided written informed consent.

Focus groups were guided by a semistructured topic guide devised using the results of the cross-sectional survey. This guide aimed to probe trainees’ clinical experiences of digital risk, assessment, and training in more depth. We discussed our draft topic guide with clinicians working with the London Digital Safety Network and made revisions based on their feedback. The final topic guide (Multimedia Appendix 2) elicited views on what participants believed online digital risk was, whether they had observed this in their clinical practice, and how they routinely managed this clinically. Prompts also explored whether participants had training in digital risk and if not, whether they would value this.

#### Analysis

Two researchers (ME and GA; both psychiatrists with mental health research experience and only one of whom had collected data) conducted an independent thematic analysis of focus group data [24,25]. An initial coding frame was developed and adjusted as themes emerged from data, generating higher order and subthemes. Any coding disagreements were discussed together with a wider research team to clarify and refine understandings.

To encourage personal reflexivity [26] and reduce the influence of theoretical or individual biases, we ensured that our research team consisted of a range of backgrounds. GA is an adult psychiatrist and an academic with interest in digital mental health. GL and RG are child and adolescent psychiatrists. RG is an expert in digital risk and is part of the board of the UK Council for Child Internet Safety and cochairs the Digital Resilience Working Group. ME is an academic adult psychiatrist with an undergraduate degree in social sciences. AP and SJ are psychiatrists and academics with an interest in sociology and social psychology. DO is an academic general adult psychiatrist and the integrated academic training lead for psychiatry, whereas AS is an older adult academic psychiatrist. NM is a social psychologist and a specialist in qualitative research methods in mental health.

## Results

### Cross-Sectional Survey

Of the 312 psychiatry trainees attending the conference, 133 (42.6%) completed the survey although only 113 (36.2%) provided their demographic and clinical information, and we included only these respondents in our analysis to allow us to compare responses by demographic characteristics. There was an approximately equal balance of men and women and of core and higher trainees (Table 2), and our sample covered a range of training grades. Trainees worked in a variety of areas across inner and suburban London (Table 2). There was a high level of digital literacy, with 92.9% (105/113) possessing a smartphone and 61.9% (70/113) reporting use of a computer or smartphone for more than an hour per day.

**Table 2:** Summary of demographic, professional backgrounds, and digital technology use.

Characteristics		Values, n (%)
**Gender**		
	Male	53 (46.9)
	Female	60 (53.0)
**Training grade**		
	Core trainee	60 (53.0)
	Higher trainee	48 (42.5)
	Others	5 (4.4)
**Experience as psychiatrist (years)**		
	0-5	64 (56.6)
	5-10	40 35.4)
	≥11	9 (8.0)
**Psychiatry specialty**		
	Children and adolescents	22 (19.5)
	Working age adults	63 (55.8)
	Older adults	11 (9.7)
	Forensic patients	11 (9.7)
	Others	7 (6.2)
**Digital literacy**		
	Possesses a smartphone	105 (92.9)
	Possesses a tablet	59 (52.2)
	Possesses a personal computer or laptop	92 (81.4)
	Uses a phone/computer for more than an hour per day	70 (61.9)

#### Digital Risk Assessment

In total, 50.4% (57/113) of the respondents reported having treated patients who had been exposed to risk related to web-based activity (Table 3). A total of 23.0% (26/113) said they had never considered the impact of patients’ *digital life* on their mental health, as part of their assessments. CAMHS trainees were not more likely to inquire about the impact of their patients’ digital lives (20/22, 91% vs 67/91, 74%; *P*=.10), despite being more likely than other specialty trainees to have previously treated patients affected by digital risk (17/22, 77% vs 40/91, 44%; *P*=.02).

Trainees most commonly reported assessing whether patients sought web-based information about their mental health problems (99/113, 87.6%) and whether they had purchased drugs or medication on the web (83/102, 81.4%). CAMHS trainees were more likely to report asking patients about specific risk domains, including reckless web behavior (18/20, 90% vs 54/82, 66%; *P*=.03), prosuicide websites (20/21, 95% vs 57/81, 70%; *P*=.01), and sexual web behavior (17/21, 81% vs 44/81, 54%; *P*=.02).

**Table 3:** Clinical experience and practice in relation to digital risk.

Question asked, responses			All trainees (n=113), n (%)	CAMHS^a^ trainees (n=22), n (%)	Other trainees (n=91), n (%)	Chi-square (*df*)	*P* value
**In your clinical work, have you treated any patients who have been exposed to risk relating to web-based activities?**						8.2 (2)	.02
		Yes	57 (50.4)	17 (77)	40 (44)		
		No	32 (28.3)	2 (9)	30 (33)		
		Unsure	24 (21.2)	3(14)	21 (23)		
**In your usual clinical practice, do you ask patients about:**							
	**The impact of their digital life**					2.9 (1)	.10
		Yes	87 (76.9)	20 (91)	67 (74)		
		No	26 (23.0)	2(9)	24 (26)		
	**Whether they access web information about mental health**					1.5 (1)	.30
		Yes	99 (87.6)	21 (95)	78 (86)		
		No	14 (12.4)	1 (5)	13 (14)		
	**How much time they spend on the web^b^**					4.2 (1)	.05
		Yes	75 (72.8)	19 (90)	56 (68)		
		No	28 (27.2)	2(10)	26 (32)		
	**Whether they engage in reckless web behavior^c^**					4.5 (1)	.03
		Yes	72 (70.6)	18 (90)	54 (66)		
		No	30 (29.4)	2 (10)	28 (34)		
	**Whether they access prosuicide websites^c^**					5.6 (1)	.01
		Yes	77 (75.5)	20 (95)	57 (70)		
		No	25 (24.5)	1 (5)	24 (30)		
	**Whether they have been a victim of cyberbullying^b^**					3.1 (1)	.06
		Yes	78 (75.7)	19 (90)	59 (72)		
		No	25 (24.3)	2 (10)	23 (28)		
	**Whether they engage in sexual web behavior^c^**					4.9 (1)	.02
		Yes	61 (59.8)	17 (81)	44 (54)		
		No	41 (40.2)	4 (19)	37 (46)		
	**Whether they buy drugs/medication on the web^c^**					0.3 (1)	.42
		Yes	83 (81.4)	18 (86)	65 (80)		
		No	19 (18.6)	3 (14)	16 (20)		

^a^CAMHS: child and adolescent mental health services.

^b^On the basis of survey responses from 103 participants.

^c^On the basis of survey responses from 102 participants.

#### Trainee Competence

Approximately two-third (76/113) of the trainees stated that they did not consider themselves competent in assessing online risk behavior; 90.3% (102/113) had not received training in digital risk assessment, and overall, 73.4% (83/113) reported that they would value this (Table 4). The majority of trainees reported using a proforma from their local hospital trust to record risk assessment (95/113, 84.1%), but only 5% (5/95) said it contained a prompt about internet use.

The trainees who had received training in assessing online risk were more likely to rate themselves as competent to assess online risk (OR 6.7, 95% CI 1.7-27.1; *P*=.01), although there was no difference in whether they reported asking patients about the impact of their digital life than those who had not been trained (OR 1.4, 95% CI 0.3-6.9; *P*=.69).

**Table 4:** Confidence and training in relation to digital risk.

Question asked, responses		All trainees (n=113), n (%)	CAMHS^a^ trainees (n=22), n (%)	All other trainees (n=91), n (%)		Chi-square (*df*)		*P* value
**Do you consider yourself competent to assess web risk?**					0.8 (1)		.25	
	Yes	37 (32.7)	9 (41)	28 (31)				
	No	76 (67.3)	13 (59)	63 (69)				
**Have you ever had any training in assessing web risk?**					2.2 (1)		.14	
	Yes	11 (9.7)	4 (18)	7 (8)				
	No	102 (90.3)	18 (82)	84 (92)				
**Would you value this training if it were offered?**					0.86 (2)		.65	
	Yes	83 (73.5)	16 (73)	67 (74)				
	No	6 (5.3)	2 (9)	4 (4)				
	Unsure	24 (21.2)	4 (18)	20 (22)				

^a^CAMHS: child and adolescent mental health services.

#### Qualitative Focus Groups

There were 11 trainees who participated in 3 focus groups. Ages ranged from 25 to 35 years, participants were all born in the United Kingdom but identified as a variety of ethnic backgrounds, and most were female. The first group comprised 5 core trainees, the second included 3 higher trainees working in general adult psychiatry, and the third included 3 higher trainees specializing in child and adolescent psychiatry.

From the analysis of transcripts, we identified 2 main themes: barriers to assessment and management of digital risk and the double-edged sword of web use.

The findings of these key themes and their subthemes are discussed as follows.

### Barriers to Assessment and Management

Most focus group participants reported that they rarely assessed digital risk when carrying out standard psychiatric risk assessments, and the first theme concerned the implied barriers to this assessment. These involved both reluctance to ask about risk and conceptual difficulties in understanding and measuring that risk.

#### Migration of the Social World

One explanation for not assessing digital risk was that their practice had not yet adapted to a recent shift of patient’s social lives to a new digital space. This was conceptualized by the CAMHS trainees as a new and distinct *third world,* which was not merely a new social space but also a *thinking space* for their patients. Participants were struck by how this migration of the patient’s social world into a more public space led to greater exposure of patients’ private thinking, blurring previous distinctions. This migration was observed particularly by the CAMHS trainees, reflecting that children and adolescents may be more likely to share private information on the web:

…the thinking space, the social world of children and adolescents has migrated.

Respondent 1 (R1) CAMHS trainee

As Child Psychiatrists we instinctively start talking to young people about school and home life, so they are the two worlds we think of… It's almost kind of getting hang of the idea that actually there's this third world.

R4 CAMHS

#### Novelty

The complexity, novelty, and pace of change of the internet and available digital tools impacted all participants’ confidence in assessing digital risk, either through a lack of technical understanding or the fear of appearing *out of touch* to their patients. All groups identified that age discrepancies between themselves and their patients created an additional challenge because of a gap in knowledge and experience. This impeded assessment as trainees felt that if they did not have firsthand experience of the digital platforms themselves, then they were less able to quantify and manage the associated risks. This was particularly marked in the CAMHS focus group but was raised to a lesser extent by the younger trainees, who were more familiar with a range of different digital tools and apps and considered that their older senior colleagues would be even less aware of new technological developments and risks:

I think it's just a fact that some of the older clinicians just don’t know about some of these apps and websites--And things that are there. So if you don’t know about it, how do you ask?

R2 CAMHS

All groups described their patients’ online interactions as having a hidden quality, which made the assessment of the extent or nature of the risk particularly challenging when contrasted with more easily quantifiable risks in the physical world, such as drug and alcohol intake or social isolation. Therefore, trainees were less likely to ask about a risk unless they felt confident that they could understand, measure, and manage that risk in an informed way:

It's always difficult these days trying to ascertain someone’s level of social support because normally you'd say how many friends do you see in an average month? But of course now it's impossible to work that out, or to really make a valid judgement of how genuine those relationships are.

R3 general adult psychiatry trainee (general)

#### Lack of Training

Participants in all groups reported a scarcity of training on how to approach digital risk assessment. None of the prompts they had embedded into their risk assessments from the start of their training included questions on digital use. Core trainees also noted the absence among colleagues of any consensus on how to ask about these risks:

*...clinicians were just told in the team meeting, oh you've just got to ask about online risk, or something like that. So actually I don’t know how individual clinicians asked about it….I don’t know. It wasn’t a “set question” kind of thing, itwas just a “do it your own way.”* [R1 CAMHS]

Despite being identified as digitally literate, core trainees expressed the most anxiety about how to word their questions and the degree to which it was appropriate to probe directly into patients’ private *online* worlds. This lack of training impacted the confidence of all participants in asking direct questions about the patient’s internet use, preferring to ask indirectly or more commonly to let patients raise it themselves:

I think if it's something that they bring up then I'd feel comfortable exploring that…But it's not something that I would consciously bring up just, you know, without them already inputting it to be honest.

R2 general

However, the intentions behind indirect questions differed slightly by years of experience. The higher clinicians reported using deliberately *vague* questions to carefully tease out more information, whereas the younger and less-experienced trainees were anxious about causing harm through suggestions. Their fear was that by assessing risk, they might paradoxically increase it:

I felt like, what if I put ideas in their head? What if they're not doing that?

R1 core trainee (core)

There is that worry...am I putting ideas into their head? But so long as it's open, so I’ll never name a specific website...I think I've approached it in quite a round-about way rather than being quite direct.

R5 core

All participants wanted more formal support and guidance in both assessing and managing any risks identified. They felt the need to adapt their existing generic risk assessments to include digital risk prompts to encourage habitual assessment. The core trainees suggested that training should also include advice about how to manage the risk, and not merely assess it. All 3 groups felt that training in the form of specific digital risk assessment workshops, vignettes, and role plays (which are teaching approaches embedded in the psychiatry curriculum for general risk assessment) would be necessary to improve confidence in assessment and management:

I think just embedding it in our training framework is a good start. When we learn how to conduct assessments in psychiatry, we have these headings. And when we learnt there was nothing around this kind of stuff, but even bringing this into part of the social history that you take. Even that as a starter will make them more conscious of it right from when they start training.

R3 general

All 3 groups identified that the combination of a lack of formal training, limited prior experience of managing these risks, and an absence of agreed-upon standards of assessment resulted in reactive rather than proactive approaches to digital risk assessment.

#### Trade-Off Between Assessment and Privacy

All trainees acknowledged a trade-off between respecting their patients’ private internet use and accurately quantifying existing digital risks. This tension was most evident in the 3 groups’ differing attitudes concerning the ethics of searching for patients on the web to assess risk. This topic arose spontaneously in all the groups with multiple participants recalling prior experiences of patient’s family members and other patients (on an inpatient unit) or the patients themselves informing medical teams of reporting of risk on social media, for example, posting about suicidal ideation.

In the core trainee and general adult groups, participants reported how they either independently searched or are encouraged, in some cases, by their team/consultant to search for patients on the web and used the information to inform risk assessments or monitor mental states via public posting on web-based platforms. Some trainees, in particular those in the CAMHS group, felt strongly that this was an unjustified invasion of privacy, which might compromise their therapeutic relationship, and was outside of their role as psychiatrists. Participants in the other groups, by contrast, largely argued that psychiatry is by definition concerned with the private information and questioned further whether information on the web could still be described as strictly private given that it is a public domain. Another tension arose here between information gathering to assess risk and the significance of the patient’s awareness of those risks. If a patient cannot identify the risks of their web use, the core trainees reasoned that they were less likely to disclose the behavior in the first place, making the search for collateral information all the more necessary:

It's on the internet, it's not like you've gone into their house and unlocked a drawer and tried to find their personal documents. It's something that anyone could, you know, in theory find.

R4 core

*But I don’t think it's our job as doctors to be looking at people’s social media and then managing...what they do. BecauseI think that's a step too far, I think that's not our role. I think if someone’s on social media and they're posting things and people who look at their social media have a responsibility to act on that. In the same way that if someone was at school and saying worrying things the people around them would have a responsibility to act on that and bring it to you.* [R1 CAMHS]

### Double-Edged Sword

The second theme was the double-edged nature of digital use, balancing risks with a range of perceived benefits for patients. Despite reporting many experiences of digital risks, such as access to prosuicide websites, all trainees felt that digital engagement brought their patients many discernible benefits by promoting social inclusion and keeping them in step with societal culture. Many were also familiar with resources for self-management, such as access to online peer support forums, various mental health apps, and the patient’s ability to seek relevant sources of information independent of service providers:

We encourage our patients to be part of the society, we avoid stigma, isolation, and we try to integrate them into the society. But the society is going (in) a different direction, and technology is being a major part of our life. We can argue whether this is all good or bad...

R4 core

#### Broadcasting Mental State

All 3 groups reported prior experience of patients using social media to broadcast their thoughts and feelings on the web. This was identified as particularly risky, with trainees describing multiple examples where this exposed patients to cyberbullying and other negative social repercussions. Concern arose particularly where repercussions were unanticipated by patients as they were frequently unwell at the time of posting. However, some trainees had experienced cases where this form of public expression had in fact acted as an alert to friends and family and to mental health professionals involved. This was either because they were explicitly or implicitly asking for help through online posting or because unusual or disturbing posts triggered referrals and interventions from family and friends:

Sometimes maybe it can be helpful if they share. They may not share with me, but they may share their suicidal thoughts on Facebook, so that will be a good alarming point for us. It will help sometimes; it may help us to pick up the risk.

R2 core

#### Anonymity

Many trainees cited examples where the anonymity of other internet users exacerbated the impact and ferocity of cyberbullying and meant that they were unable to address this. They perceived that people were often more disinhibited on the web owing to the lack of accountability or repercussions. However, anonymity was also cited as a benefit for patients who find it difficult to talk about mental health problems in person. They were able to find a forum on the web where they could discuss sensitive issues protected by their anonymity:

I think people are just a lot more disinhibited online. And I think that can be a good thing because it might mean that someone will open up about something that they would not speak to somebody about in person...But then that can also be a bad thing because people then might be horrible in ways that they would never be in person, if you post something someone could be really, really unpleasant about you in a really upsetting hurtful way, which then has an impact on your mental health. And it stays there...forever.

R3 CAMHS

#### Vulnerability

Although the use of the internet poses potential risks to any individual, trainees were concerned that their patients were particularly vulnerable for reasons of diminished capacity and emotional instability. Exposure to abuse or risky behaviors on the web could lead to extreme risks for someone who was unwell and/or had impaired ability to make proportionate or safe decisions:

One of the patients had her ex-partner put up sexually inappropriate material of her on Facebook, and she wasn’t in a position where she could deal with that. And actually that made her suicidal ideation significantly worse. And there have been psychotic patients who have misinterpreted things on, you know, the apps like Tinder and meeting apps. And that puts them in a lot of risk as well because they end up meeting people in very vulnerable situations.

R1 core

#### A Life Curated

The CAMHS group warned that the opportunity to construct a curated online life on social media sites can create pressures on adolescents to conceal difficulties and fixate on unachievable goals of perfectionism. This discrepancy between what is real and what is on the web can undermine the potential benefits of social media sites, reinforcing patients’ low self-esteem and exacerbating existing mental health problems. It can also prevent patients from forming rewarding social connections in which they feel able to confide in their peers about their difficulties. The benefit, however, of controlling what the world can see is that the internet can be one place where people may not know that someone is unwell and where they can exist free from the stigma and the judgment of others:

there's almost like this real-life image and there's the cyber image, and how they're portrayed inside of there. And how they're perceived by others in this cyber world.

R2 CAMHS

#### Web-Based Groups

All 3 groups discussed their patients’ use of online forums for support and information sharing. These interactions had both benefits and risks. The trainees reported examples of suicide pacts formed on the web or patients using groups for advice about how to lose weight, often termed *pro-ana websites.* They expressed concern that patients might be influenced by exposure to competitive dieting and methods to conceal self-harm or be drawn into imitating the habits of people they meet on the web. These risks were tempered by an acknowledgment of the significant benefit that patients reported from online peer support forums, which were accessible whenever and wherever they needed. For patients who experienced a particular crisis at night, this was particularly important as a source of support.

## Discussion

### Principal Findings

In this first mixed methods study investigating psychiatrists’ experience of assessing their patients’ digital risk, we found that there was widespread awareness among psychiatry trainees of patients’ digital risks but variation in the depth of their assessment in routine practice. Despite the fact that child and adolescent trainee psychiatrists have more exposure to online risks, they are not more likely to ask patients about the impact of their digital lives. They were, however, more likely to ask about specific areas of risk, such as sexualized web behavior and accessing suicide websites.

An in-depth exploration of this issue through focus group interviews found that explanations for the discrepancy between awareness of risk and the tendency not to assess it routinely and comprehensively are related to anxieties over transgressing boundaries, lack of training, and the fear of repercussions of asking these questions. Trainees reported a lack of confidence in knowing what specific question to ask and whether this was acceptable and expressed concerns about potential boundary violations that may occur if searching for information about their patients online. Our study also revealed the extent to which trainees value their patients’ access to digital tools. Trainees saw digital engagement as an opportunity to reduce social isolation and an opportunity to enhance self-management and recovery in keeping with the current direction of modern mental health care.

To date, there is a paucity of research on digital risk assessment in psychiatry. However, the fear of the negative impact of asking questions about harmful use of the internet is analogous to the widespread belief that asking about suicidal ideation increases the risk of suicide attempts, which has been discredited [27]. Most participants lacked training in asking questions related to digital risk but stated that they would value this; there is potential that greater experience in assessing this domain may ameliorate the concerns about the harmful impact of assessment.

The uncertainty over the ethics, legality, and impact of searching for one’s patients on the internet have been discussed in the literature [28,29]. In our study, the ethics of researching the online behavior of patients when disclosure is not forthcoming appeared to pivot on patients’ awareness of risks, but different trainees interpreted the ethical significance differently. Some argued that if the patient intended the information to be public, then there was no violation, but if they were unaware of its public nature, either through illness or lack of capacity, then the information should be regarded as private, the assumption being that the patient’s awareness or lack thereof has the power to define the status of the information. Conversely, other trainees saw unknown or misunderstood public exposure of intended private data as part of the digital risk assessment itself and something they should be striving to protect their patients from. Both positions therefore find justification through intention; one seeks to define what is private through what is intended as private and the second defines risk where intention is absent. This example reveals the struggle to understand where these new lines should be drawn and what a psychiatrist’s role should be within this new paradigm. Where does a psychiatrist’s responsibility to the patient’s mental health end and their right to privacy begin?

### Strengths and Limitations

To our knowledge, this is the first study to explore attitudes toward digital risk among psychiatry trainees. Our mixed methods approach allowed us to gain both breadth and depth in our analysis, with the quantitative findings informing the qualitative phase. We sampled from a large metropolitan area and achieved wide participation in our focus groups. The main limitation of our study is the generalizability of findings to psychiatrists in settings beyond the United Kingdom. However, we are not aware of any similar studies done in any other countries, and given the high internet use in the United Kingdom and rising use of the internet internationally, we would strongly encourage mental health clinicians in other countries to take note of our findings, do similar surveys, and develop their digital risk guidance tailored to their population. The response rate was relatively low, despite efforts to survey all attendees of the conference both through an electronic and paper copy. As there is no published questionnaire for assessing experiences of digital risk, we developed our own tool, using a multidisciplinary team of digital experts and extensive piloting, but we were not able to validate this. Our qualitative study responses may have been influenced by social desirability bias, as focus groups consisted partly of colleagues who worked together. This led to the suppression of any opinions, which deviated from apparent social norms, thereby censuring the debate. Finally, these data, from 2013 to 2016, may not reflect current practice and experience as digital literacy may have increased, but digital risk may also have increased. Given the lack of other studies on this topic, our study represents the best available evidence.

### Conclusions

Our study identified generally high levels of self-reported awareness among psychiatry trainees of internet-related risk behavior, but we also found wide variation in their confidence in assessing this risk in routine practice and barriers to assessment of digital risk. Future research should aim to assess the generalizability of these findings to clinicians working in other mental health settings and track changes in experience as well as the effectiveness of measures to improve awareness of digital risk. Our findings have important implications for clinical practice and policy. Professionals working in mental health services need to evolve their practice in line with the technological revolution. In the United Kingdom, the Department for Digital, Culture, Media, and Sport has published a strategy for web safety that is underpinned by 3 principles: (1) what is unacceptable offline should be unacceptable online, (2) all users should be empowered to manage online risks and stay safe, and (3) technology companies have a responsibility to their users. They have developed a legislative framework for hate crime and an action plan for older people and are working closely with the Department for Education to ensure that schools have the support and resources to support children, parents, and carers [30]. MindEd, a free educational resource for teachers and parents addressing children and young people's mental health, has training modules on online risk and resilience and online safety and well-being [31]. The Information Commissioner's Office, a nondepartmental public body, has written a code of practice for age-appropriate design of web services that is subject to parliamentary approval [32]. Beyond these resources, digital risk must be acknowledged by clinical professional and regulatory bodies, and we recommend that the Royal College of Psychiatrists and Health Education England lead on providing training on digital risk assessment and ethical frameworks for clinicians on researching web behavior of their patients and that similar approaches are adopted in other countries. Digital risk assessment should be embedded in policies and procedures for mental health trusts so that they become usual practice, so that clinicians at all stages of training learn about enhanced risk assessment and from risk incidents. Digital literacy is an important skill for the future of health services, and there is an urgent need to promote good clinical practice and prevent a potential blind spot in psychiatric risk assessment.

## Appendix

Multimedia Appendix 1 Questionnaire.

Multimedia Appendix 2 Topic guide.

## Abbreviations

CAMHS: child and adolescent mental health services
NHS: National Health Service
