# Supplementary material for: Assessing Digital Risk in Psychiatric Patients: Mixed Methods Study of Psychiatry Trainees’ Experiences, Views, and Understanding
Source: JMIR Ment Health. 2020 Jul 29;7(7):e19008. doi: 10.2196/19008 (PMC7424482; doi:10.2196/19008)
Supplement: Multimedia Appendix 1 [file mental_v7i7e19008_app1.doc]

**Assessment in the Digital Age**

We are two psychiatry trainees interested in exploring current approaches to clinical risk assessment in the digital age, when almost all service users will to a greater or lesser extent use digital technology particularly internet and mobile phones (i.e. on-line activity). We are conducting a simple survey amongst trainee psychiatrists in order to get a better understanding of the extent to which professionals consider the service user’s use of digital technology.

We would be very grateful if you would take a few minutes to complete this anonymous survey. If you would like to receive feedback following the conclusion of our study please include your email address at the end of the survey.

Please direct any queries on this survey to g.aref@ucl.ac.uk

***Section 1***

**a) In your current practice have you ever considered (i.e. have in mind) the impact of a service user's ‘digital life’ i.e. use of internet/social media on their mental health?**

YES ☐ NO ☐

**b) In your current practice do you consider whether service users access information on-line or via social media, in relation to their mental health problems?**

YES ☐ NO ☐

*(if yes please continue below, if no to BOTH a) and b) please go to section 4)*

***Section 2***

**How often, when seeing service users in the following age groups do you consider your service user’s on-line activity?**

**7-18yrs**

In all cases☐ In most cases ☐ In a few cases ☐ Never ☐ Not applicable☐

**19-64 yrs**

In all cases☐ In most cases ☐ In a few cases ☐ Never ☐ Not applicable☐

**65+yrs**

In all cases☐ In most cases ☐ In a few cases ☐ Never ☐ Not applicable☐

***Section 3***

**In your current practice when conducting an assessment of your service users do you ask about:**

**Length of time engaged in on- line activity/social media**

In all cases☐ In most cases ☐ In a few cases ☐ In one or two cases ☐ Never☐

**Seeking information about their mental health problems on-line**

In all cases☐ In most cases ☐ In a few cases ☐ In one or two cases ☐ Never☐

**Exploring treatment options and other forms of help for their mental health problem on-line**

In all cases☐ In most cases ☐ In a few cases ☐ In one or two cases ☐ Never☐

**Acting recklessly on-line following mental health deterioration**

In all cases☐ In most cases ☐ In a few cases ☐ In one or two cases ☐ Never☐

**Accessing on-line website or forums which may promote activities harmful to self** *(e.g.: pro-anorexia or pro-mia)*

In all cases☐ In most cases ☐ In a few cases ☐ In one or two cases ☐ Never☐

**Accessing forums which may promote suicide** *(e.g. pravada-painless ways to commit suicide)*

In all cases☐ In most cases ☐ In a few cases ☐ In one or two cases ☐ Never☐

**On-line bullying (either victim or perpetrator)**

In all cases☐ In most cases ☐ In a few cases ☐ In one case ☐ Never☐

**Sexual on-line behaviour such as accessing pornography, or ‘grooming’ or ‘sexting’** (*Sexting is the act of sending sexually explicit messages or photographs, primarily between mobile phones.)*

In all cases☐ In most cases ☐ In a few cases ☐ In one or two cases ☐ Never☐

**Use of gambling websites**

In all cases☐ In most cases ☐ In a few cases ☐ In one or two cases ☐ Never☐

**Obtaining non- prescription medication on-line**

In all cases☐ In most cases ☐ In a few cases ☐ In one or two cases ☐ Never☐

***Section 4***

**Have any of the service users you have seen in the past 6 months talked about their on-line behaviour without prompting or questioning?**

A few of my patients☐ Some of my patients☐ Lots of my patients ☐ No ☐

**In your current work do you use a standardised risk pro forma to record your risk assessment?**

Yes ☐ Sometimes ☐ No ☐

**Does this pro forma contain a section to record on-line risk behaviours?**

Yes ☐ No☐ Unsure ☐ Not applicable☐

**In your clinical work have you assessed or treated any service users who have been exposed to risk relating to on-line activity?**

Yes ☐ No☐ Unsure ☐

**If ‘Yes’ it would be helpful if you could add a few (anonymised) details (optional)**

**Do you consider yourself competent to assess on-line risk?** Yes ☐ No☐

**Please feel free to add any comments you may have about this (optional)**

**Have you ever had any training in on-line risk assessment?**

Yes ☐ No☐

**Would you value training in this area if it could be offered?**

Yes ☐ No ☐ Unsure ☐

# Section 5

**About your own digital media experience:**

**Do you have?**

Smartphone ☐ Tablet ☐ PC/Laptop ☐ None of the above ☐

**Do you use these sites?**

Facebook ☐ Twitter ☐ YouTube ☐ On-line news ☐

Gaming ☐

Other ☐ please specify_______________________

**How often do you use mental health websites as a resource to access information?**

**How often do you recommend these websites to service users?**

Never ☐ Rarely ☐ Sometimes ☐ Often ☐

Never ☐ Rarely ☐ Sometimes ☐ Often ☐

**What Mental Health websites do you recommend? (free text box-optional)**

**Would you find it helpful to have a list of NHS approved mental health websites that may assist you and service users?**

Yes ☐ No ☐

**Approximately how often do you go on-line on your phone/computer each day? This is how often you access them on purpose, for any activity, whether gaming, messaging, checking email etc.**

More than 10 times an hour☐

5-10 times per hour☐

Up to 5 times per hour☐

Less than once per hour☐

**How much is your on-line life (emails / Facebook) on your mind? That is, you think about it a lot and it is hard to stop thinking about it?**

Never ☐ Rarely ☐ Sometimes ☐ Often ☐ Always ☐

**Do you worry that you use the internet too much?**

Never ☐ Rarely ☐ Sometimes ☐ Often ☐ Always ☐

**Does time spent on the internet get in the way of your work?**

Never ☐ Rarely ☐ Sometimes ☐ Often ☐ Always ☐

**Does time spent on the internet get in the way of your social and family life?**

Never ☐ Rarely ☐ Sometimes ☐ Often ☐ Always ☐

**A few details about you: Your gender : Male ☐ Female ☐**

| **Current position** | | |  |
| --- | --- | --- | --- |
| CT1-3 ☐ |  |  |  |
| ST4-7 ☐ |  | Other (please specify)___________________________ |  |

**How many years’ experience do you have in your current profession?**

0-5 ☐ 6-10☐ 11-19 ☐ 20-30 ☐ More than 40 ☐

**Training Scheme**

| UCL Partners ☐ |
| --- |
| South London and The Maudsley ☐ |
| East London NHS Foundation Trust ☐ |
| North East London NHS Foundation Trust ☐ |
| South West London and St George’s Mental Health Trust ☐ |
| Charing Cross Charing Cross |
| St Mary’s ☐ |
| Other please specify ………………………………………………………………… |
| Not applicable |

**What service user group do you currently work with?**

Child and adolescent ☐

Adult ☐

Older adults ☐

Forensic ☐

Learning Difficulties ☐

Other- please specify: _________________________________________

**Please feel free to add any comments you may have about risk assessment of on line activity.**

*Thank you for completing this questionnaire*
