# Supplementary material for: Assessing Digital Risk in Psychiatric Patients: Mixed Methods Study of Psychiatry Trainees’ Experiences, Views, and Understanding
Source: JMIR Ment Health. 2020 Jul 29;7(7):e19008. doi: 10.2196/19008 (PMC7424482; doi:10.2196/19008)
Supplement: Multimedia Appendix 2 [file mental_v7i7e19008_app2.doc]

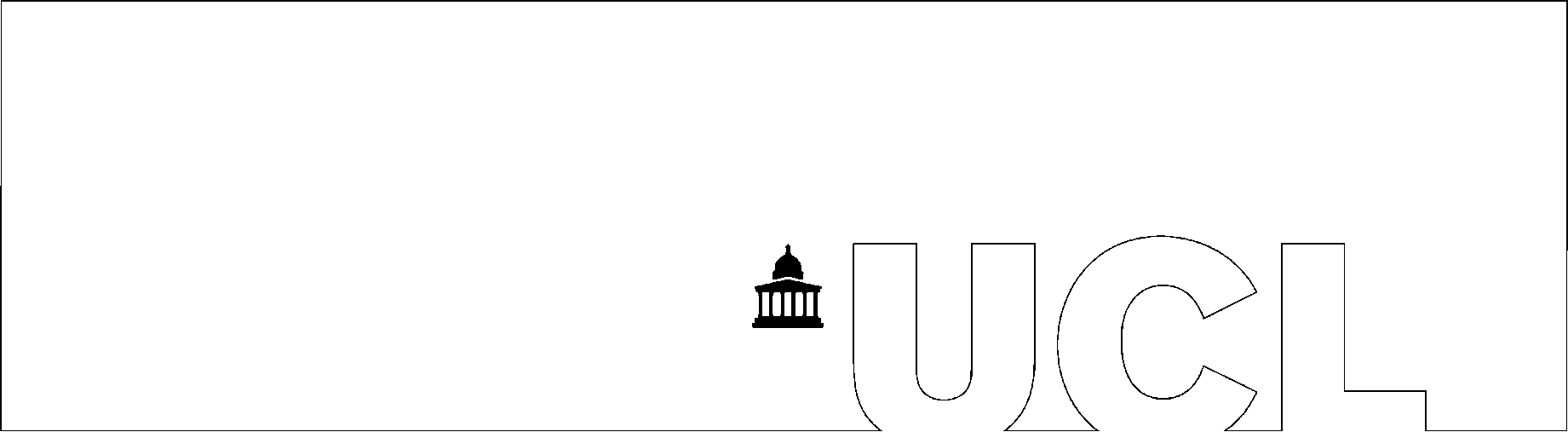
 **Psychiatry trainees’ views and experiences of digital risk in psychiatry**

**Topic Guide for Staff**

**Introductory discussion about the aims of the project**

**Theoretical importance of digital risk in psychiatry**

**What is digital risk in psychiatry and how can this affect our patients?**

**What is online digital risk?**

**Can you think of any patients in whom you or another team member found their digital life impacted on their mental health?**

- **How was it discovered?**
- **What was the outcome and management?**

**How does it usually present?**

**What aspects of digital risk can be important in psychiatry?**

**Does the importance of digital risk vary amongst the different specialities with psychiatry?**

**To what extent does psychiatric risk assessment assess people’s digital risk to themselves and others at present?**

**What questions do they ask? What is this based on?**

**How might professionals pick up digital risk more effectively?**

**Have they had any digital risk assessment training and would they value this?**
